# Exploring novel immunotherapy in advanced esophageal squamous cell carcinoma: Is targeting TIGIT an answer?

**DOI:** 10.1007/s10388-024-01105-4

**Published:** 2025-01-23

**Authors:** Chien-Huai Chuang, Jhe-Cyuan Guo, Ken Kato, Chih-Hung Hsu

**Affiliations:** 1 Department of Medical Oncology, National Taiwan University Cancer Center, 7 Chung-Shan South Road, Taipei, 10002 Taiwan; 2 Department of Oncology, National Taiwan University Hospital, Taipei, Taiwan; 3 Graduate Institute of Oncology, National Taiwan University College of Medicine, Taipei, Taiwan; 4 Department of Head and Neck, Esophageal Medical Oncology, National Cancer Center Hospital, 5-1-1 Tsukiji, Chuo-Ku, Tokyo, 104-0045 Japan; 5 Department of Gastrointestinal Medical Oncology, National Cancer Center Hospital, Tokyo, Japan

**Keywords:** Immunotherapy, T-cell immunoreceptor with Ig and ITIM domains, Esophageal squamous cell carcinoma; Immune checkpoint inhibitor

## Abstract

Esophageal squamous cell carcinoma (ESCC) is a prevalent and highly lethal malignancy in Asia. Recent advancements in immune checkpoint inhibitors (ICIs) have markedly transformed the systemic therapy landscape for ESCC. Anti-PD-1-based combination with chemotherapy or with ipilimumab, an anti-CTLA-4 antibody, have been established as the new standard first-line treatments for patients with advanced ESCC. Moreover, anti-PD-1 monotherapy has demonstrated improved efficacy and survival compared with second-line chemotherapy in previously treated patients with ESCC. Novel ICIs targeting other immune checkpoints also show potential for enhancing anticancer therapy in advanced ESCC.The TIGIT/PVR pathway represents a new immune checkpoint. Preclinical studies have indicated that the dual blockade of TIGIT and PD-1 can enhance antitumor immune responses. Clinical trials have reported that combining anti-TIGIT with anti-PD-1/PD-L1 antibodies elicited clinical responses in patients with advanced ESCC. In the first-line systemic therapy setting, combinations of dual ICIs targeting TIGIT and PD-1/PD-L1 plus platinum-based chemotherapy have demonstrated acceptable toxicity profiles and promising antitumor activity in several phase II trials and one phase III study. However, the role of adding an anti-TIGIT antibody to the current standard of anti-PD-1/PD-L1 plus platinum-based chemotherapy in first-line therapy for advanced ESCC remains to be fully determined, necessitating further clinical trials. Ongoing studies are also investigating the role of anti-TIGIT, with or without anti-PD-1/PD-L1, in locoregional ESCC. Additional research is essential to optimize the potential of anti-TIGIT therapy in ESCC and other malignancies by identifying predictive biomarkers, determining optimal antibody types, and gaining key mechanistic insights.

**Supplementary Information:**

The online version contains supplementary material available at 10.1007/s10388-024-01105-4.

## Current treatment landscape of advanced esophageal squamous cell carcinoma

Esophageal cancer (EC) is the seventh most common and sixth most lethal malignancy globally [1]. Over 80% of the global disease burden is concentrated in Asian countries, where esophageal squamous cell carcinoma (ESCC) is the most prevalent histologic type. ESCC differs from esophageal adenocarcinoma, which is relatively common in Western countries, in terms of etiology, carcinogenesis, epidemiology, and clinical presentations [2].

Several phase III studies have demonstrated that immune checkpoint inhibitors (ICIs) targeting the programmed cell death protein 1 (PD-1)/programmed cell death ligand 1 (PD-L1) pathway are more effective than second-line chemotherapy for previously treated patients with advanced ESCC [3, 4]. Recently, multiple phase III studies have compared the efficacy and safety of an anti-PD-1/PD-L1 ICI combined with platinum-based chemotherapy vs. chemotherapy alone as the first-line systemic therapy for patients with unresectable locally advanced or metastatic ESCC [5–12]. As summarized in Table 1, these studies have consistently demonstrated substantial improvements in the objective response rate (ORR), progression-free survival (PFS), and overall survival (OS), favoring the combination of anti-PD-1/PD-L1 with chemotherapy. The combination of an anti-PD-1/PD-L1 ICI with chemotherapy has thus been established as the new global standard for first-line systemic therapy in advanced ESCC. Moreover, a chemotherapy-free dual immune checkpoint blockade, combining nivolumab (targeting PD-1) with ipilimumab (targeting cytotoxic T-lymphocyte-associated protein 4), considerably prolonged OS compared with chemotherapy alone used as the first-line systemic treatment for patients with advanced ESCC [6]. Collectively, the unprecedented success of ICIs in reshaping the new treatment landscape for advanced ESCC indicates the need for further exploration of novel immunotherapies, either as first-line combination therapies or as subsequent options after progression on earlier ICI treatments.

**Table 1:** Key Results of Phase 3 Trials of Anti-PD-1/PD-L1 Therapy-based Combination vs. Chemotherapy as First-Line Systemic Therapy for Advanced Esophageal Squamous Cell Carcinoma

Trial	Population	Histology	P’t No	Treatment	Median OS (95% CI) (months)	HR (95% CI)	*P* value
Anti-PD-1/PD-L1 ICI plus chemotherapy vs. Chemotherapy							
KEYNOTE-590							
	ESCC subgroup	SCC 100%	274	Pembrolizumab + Chemotherapy	12.6 (10.2–14.3)	0.72 (0.60–0.88)	0.0006
			274	Chemotherapy	9.8 (8.6–11.1)		
CheckMate-648							
	All randomized patients	SCC 100% AC 0%	321	Nivolumab + Chemotherapy	13.2 (11.1–15.7)	0.74 (0.58–0.96)	0.002
			324	Chemotherapy^a^	10.7 (9.4–11.9)		
ESCORT-1st							
	All randomized patients	SCC 100% AC 0%	298	Camrelizumab + Chemotherapy	15.3 (12.8–17.3)	0.70 (0.56–0.88)	0.001
			298	Chemotherapy^a^	12.0 (11.0–13.3)		
ORIENT-15							
	All randomized patients	SCC 100% AC 0%	327	Sintilimab + Chemotherapy	16.7 (14.8–21.7)	0.63 (0.51–0.78)	< 0.0001
			332	Chemotherapy^a^	12.5 (11.0–14.5)		
			193	Chemotherapy	13.6 (11.3–15.7)		
JUIPITER-06							
	All randomized patients	SCC 100% AC 0%	257	Toripalimab + Chemotherapy	17.0 (14.0-NR)	0.58 (0.43–0.78)	< 0.001
			257	Chemotherapy^a^	11.0 (10.4–12.6)		
RATIONALE-306							
	All randomized patients	SCC 100% AC 0%	326	Tislelizumab + Chemotherapy	17.2 (15.8–20.1)	0.66 (0.54–0.80)	< 0.001
			323	Chemotherapy^a^	10.6 (9.3–12.1)		
ASTRUM-007							
	All randomized patients (PD-L1 CPS ≥ 1)	SCC 100% AC 0%	368	Serplulimab + Chemotherapy	15.3 (14.0–18.6)	0.68 (0.53–0.87)	0.002
			183	Chemotherapy^a^	11.8 (9.7–14.0)		
GEMSTONE-304							
	All randomized patients	SCC 100% AC 0%	358	Sugemalimab + Chemotherapy	15.3 (13.3–17.1)	0.70 (0.55–0.90)	0.0076
			182	Chemotherapy^a^	11.5 (9.9–13.4)		
Anti-PD-1 plus anti-CTLA4 (dual ICIs) vs. Chemotherapy							
CheckMate-648 (dual ICIs vs. chemotherapy)							
	All randomized patients	SCC 100% AC 0%	325	Nivolumab + ipilimumab	12.8 (11.3–15.5)	0.78 (0.62–0.98)	0.011
			324	Chemotherapy^a^	10.7 (9.4–11.9)		

*AC* adenocarcinoma, *CI* confidence interval, *CPS* combined positive score, *CTLA4* cytotoxic T-lymphocyte antigen 4, *HR* hazard ratio, *No* number, *OS* overall survival, *PD-L1* programmed cell death protein ligand-1, *SCC* squamous cell carcinoma

In addition to PD-1 and CTLA4, multiple cell surface coinhibitory receptors have been identified to play crucial roles in regulating T-cell activation, thus emerging as potential therapeutic targets for the development of novel ICIs [13, 14]. An ICI targeting lymphocyte-activation gene 3, another such coinhibitory receptor, has recently been approved for the treatment of advanced melanoma [15, 16]. ICIs targeting T-cell immunoreceptor with Ig and ITIM domains (TIGIT; also called WUCAM, Vstm3, and VSIG9) have been actively examined in multiple cancer types, including ESCC.

In this review, we will briefly introduce the biology of TIGIT, discuss the importance of TIGIT expression in ESCC, and summarize the clinical development of anti-TIGIT ICIs in patients with ESCC.

## Brief introduction of tigit immunobiology

First identified in 2009, TIGIT is a receptor within the immunoglobulin (Ig) superfamily [17]. TIGIT consists of an extracellular Ig variable domain, a type 1 transmembrane domain, and a cytoplasmic tail, which includes an immunoreceptor tyrosine-based inhibitory motif (ITIM) and an Ig tail-tyrosine-like motif. TIGIT is expressed on activated CD8 + T cells, CD4 + T cells, natural killer (NK) cells, and regulatory T cells (Tregs) and functions as an inhibitory regulator that modulates both adaptive and innate immune responses. Preclinical studies have demonstrated that TIGIT plays a critical role in restraining T-cell-mediated inflammation.

**Fig. 1:**
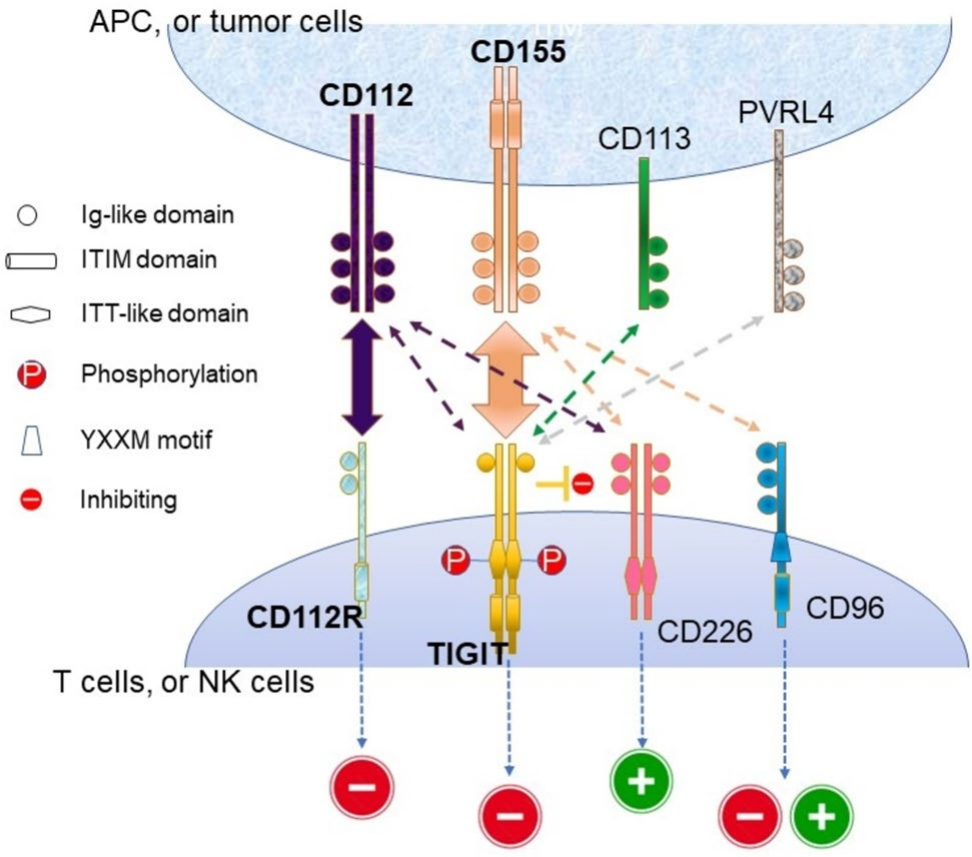
The interaction of TIGIT family receptors and ligands. Abbreviations: APC, antigen presenting cells; Ig, immunoglobulin; ITIM, immunoreceptor tyrosine-based inhibitory motif; ITT, immunoglobulin tail tyrosine; TIGIT, T cell immunoreceptor with Ig and ITIM domains; PVRL4, poliovirus receptor-related 4. Interactions between receptors and ligands are illustrated with two-sided arrows. The thickness of these arrows is proportional to the reported affinities, indicating the strength of each interaction

Figure 1 illustrates the key components of the TIGIT pathway. TIGIT and CD226, both expressed on immune cells, interact with their ligands, particularly CD155 (also known as the poliovirus receptor [PVR]), to mediate different biologic effects on immune-cell functions. When CD226 binds with its ligands, it promotes T-cell activation. By contrast, the binding of TIGIT to its ligand transduces signals that inhibit T-cell function. TIGIT has a higher affinity for its ligand than CD226 and, together with other mechanisms, it counteracts CD226-mediated signaling and leads to a predominantly immunosuppressive T-cell phenotype [18]. In the tumor microenvironment, Tregs expressing a higher level of TIGIT display a more immunosuppressive and activated phenotype [19]. In addition, high TIGIT expression is associated with the exhaustion of tumor-infiltrating NK cells. [20]

TIGIT and PD-1 are co-expressed on tumor-infiltrating lymphocytes (TILs) in both human and experimental murine cancers [21]. Although PD-1 and TIGIT are believed to regulate distinct coinhibitory receptors, studies have indicated that the inhibition of either PD-1 or TIGIT in preclinical tumor models was less effective in the absence of CD226. PD-1 inhibits the phosphorylation of both CD226 and CD28 through its ITIM-containing intracellular domain (ICD). By contrast, TIGIT’s ICD is dispensable because TIGIT impedes CD226 co-stimulation by obstructing its interaction with CD155. Full restoration of CD226 signaling and optimization of antitumor CD8 + T-cell responses may necessitate a dual blockade of both TIGIT and PD-1 [22]. Dual blockade of TIGIT and PD-1 in preclinical models has been found to enhance the antitumor immune response and improve tumor control. A recent in-depth mechanistic study revealed that an anti-TIGIT antibody enhances PD-L1 blockade, facilitating the shift of anti-tumor CD8 + T cells from an exhausted effector-like state to a more memory-like state through myeloid and Treg cells. [23]

## Significance of TIGIT expression in human ESCC

In a retrospective study using formalin-fixed, paraffin-embedded blocks from 58 patients with ESCC, high TIGIT expression—assessed through mRNA expression or immunohistochemistry (IHC) staining of TILs—was associated with unfavorable clinical outcomes [24]. In another study involving 154 patients with ESCC who underwent surgical resection without prior systemic therapy, the high expression of TIGIT alongside PD-L1 in ESCC tumors was correlated with poor survival outcomes [25]. In addition, a study using peripheral blood from a small cohort of patients with surgically resected ESCC found a positive correlation between the expression levels of PD-1 and TIGIT, as demonstrated by flow cytometry and IHC staining. [26] Further analysis of the cBioPortal Cancer Genomics and Oncomine datasets revealed that the increased expression levels of CD155, the ligand for TIGIT, were associated with poor prognosis [27]. Collectively, these findings support the coordinated expression of the TIGIT/PVR and PD-1/PD-L1 pathways in ESCC tumor tissues and identify high expression of TIGIT/PVR as a poor prognostic marker in patients with ESCC.

Chemoradiotherapy (CRT), employed either as neoadjuvant or definitive therapy, is widely used for treating locally advanced ESCC. A preclinical study demonstrated that irradiation could increase CD155 expression in ESCC cell lines. Moreover, the knockdown of CD155 not only inhibited tumor growth and migration but also enhanced radiosensitivity [27]. This finding suggests a potential interaction between anticancer therapies, such as irradiation, and immune evasion mechanisms mediated by the overexpression of immune checkpoints. Furthermore, this finding highlights the potential for improved efficacy of CRT in patients with ESCC when combined with ICIs.

## Clinical development of TIGIT-targeting agents in ESCC

TIGIT-targeting antibodies, used either alone or in combination with anti-PD-1/PD-L1 antibodies, have been explored in various cancer types, including esophageal cancer (EC). Anti-TIGIT monotherapy phase 1 trials for solid tumors, including GO30103 investigating tiragolumab and MK-7684–001 investigating vibostolimab, demonstrated limited efficacy, with objective response rates of 0–3%. Common treatment-related adverse events (TRAEs) included fatigue, pruritus, and rash, predominantly grade 1–2 (Supplemental Table S1). These results suggest that while anti-TIGIT antibodies are generally well-tolerated, their standalone efficacy as late-line systemic therapy remains modest. Regarding TIGIT-targeting antibodies in combination with anti-PD-1/PD-L1 antibodies, two early-phase clinical trials, GO30103 and AdvanTIG-105 which investigated ociperlimab, have shown promising results. These studies have demonstrated that combining an anti-PD-1/PD-L1 antibody with an anti-TIGIT antibody could induce clinical responses in patients with advanced solid tumors, including EC, who have previously undergone standard treatments (Table 2). Notably, the GO30103 trial enrolled immunotherapy-naïve patients, whereas the AdvanTIG-105 trial included patients who had previously received ICI therapy. [28, 29]

**Table 2:** Key results of trials of anti-tigit combination therapy as late-line systemic therapy for esophageal cancer

Trial	Population	Histology		P’t No	Treatment	ORR (%)	DCR (%)	Median DoR (months) (95% CI)	Median PFS (months)	Median OS (months) (95% CI)
Anti-PD-1/PD-L1 ICI plus Anti-TIGIT										
GO30103										
	Dose-expansion EC cohort	SCC 61.1% AC 38.9%		18	Tiragolumab + Atezolizumab	28	50	15.2 (7.0-NR)	Not reported	Not reported
AdvanTIG-105										
	All solid tumor patients	GC/GEJC of 3 patients No ESCC		30	Ociperlimab + Tislelizumab	10	50	3.6 (2.8-NR)	Not reported	Not reported
AdvanTIG-203										
	All randomized patients	SCC 100% AC 0%	62		Ociperlimab + Tislelizumab	30.6	61.3	Not reported	3.4	10.1 (7.1-NR)
			63		Placebo + Tislelizumab	20.7	58.7	Not reported	3.5	9.3 (6.0-NR)

*CI* confidence interval, *No* number, *OS* overall survival, *PFS* progression free survival, *DoR* duration of response, *PD-L1* programmed cell death protein ligand-1, *TIGIT* T cell immunoreceptor with Ig and ITIM domains, *SCC* squamous cell carcinoma, *AC* adenocarcinoma, *GC/GEJC* gastric/gastroesophageal junction cancer, *ORR* objective response rate, *DCR* disease control rate, *NR* not reached

The AdvanTIG-203 study, a randomized phase II clinical trial, focused on patients with advanced ESCC who had progressed after first-line platinum-based chemotherapy and had not received prior ICIs. These patients’ tumors exhibited PD-L1 expression with a tumor area positivity (TAP) of 10% or more. A total of 125 patients were enrolled and received either tislelizumab (an anti-PD-1 antibody) plus ociperlimab (an anti-TIGIT antibody) or tislelizumab plus placebo. The combination of tislelizumab and ociperlimab achieved an investigator-assessed ORR of 30.6% compared with 20.6% for the tislelizumab plus placebo group. PFS was similar between the two groups, whereas OS data remained immature (Table 2). The combination of tislelizumab and ociperlimab was generally well-tolerated with manageable toxicity. [30]

For first-line systemic treatment of advanced or metastatic EC, the combination of tiragolumab (an anti-TIGIT antibody), atezolizumab (an anti-PD-L1 antibody), and chemotherapy has been evaluated in the SKYSCRAPER-08 and MORPHEUS-EC trials (Table 3). SKYSCRAPER-08, a phase III placebo-controlled trial conducted in Asia, randomized patients with advanced ESCC into two groups: one receiving tiragolumab plus atezolizumab combined with cisplatin/paclitaxel chemotherapy and the other receiving placebos with cisplatin/paclitaxel chemotherapy. The study reported a statistically significant improvement in PFS and OS with the tiragolumab/atezolizumab chemotherapy combination compared with the placebo group. However, the absence of a treatment arm with anti-PD-1/PD-L1 plus chemotherapy makes it unclear how much tiragolumab contributes to the efficacy of this immunochemotherapy regimen. MORPHEUS-EC, a randomized phase II trial, enrolled EC patients into three treatment groups: tiragolumab plus atezolizumab with cisplatin/5-fluorouracil (5-FU) chemotherapy, atezolizumab with cisplatin/5-FU chemotherapy, and cisplatin/5-FU chemotherapy alone. Out of 152 patients with EC enrolled, 107 (70.4%) had ESCC. The results indicated a higher ORR and a numerically longer median PFS when more ICIs were added to chemotherapy, suggesting an added benefit of combining tiragolumab with atezolizumab and chemotherapy as a first-line therapy for advanced EC [31, 32]. Although TRAEs were slightly increased in the tiragolumab/atezolizumab plus chemotherapy group, they were generally manageable and did not present unexpected safety concerns.

**Table 3:** Key results of trials of anti-pd-1/pd-l1 plus anti-tigit and chemotherapy combination as first-line systemic therapy for advanced esophageal squamous cell carcinoma

Trial	Population	Histology	P’t No	Treatment	ORR (%)	Median DoR (months) (95% CI)	Median PFS (months) (95% CI)	Median OS (months) (95% CI)	Grade 3–4 AEs (%)
MORPHEUS-EC									
	All randomized patients	SCC 70% AC 30%	63	Tiragolumab + Atezolizumab + chemotherapy^a^	67.7	7.2 (4.4–15.3)	6.9 (6.0–9.9)	16.0 (11.3–21.7)	79.0
			65	Atezolizumab + chemotherapy^a^	53.8	6.9 (5.3–7.8)	6.8 (5.6–8.3)	13.1 (12.2–16.3)	80.0
			24	chemotherapy^a^	47.8	5.9 (2.8-NR)	4.1 (2.7–7.6)	9.9 (7.6–23.2)	73.9
SKYSCRAPER-08									
	All randomized patients	SCC100%	226	Tiragolumab + Atezolizumab + chemotherapy^b^	59.6	7.1 (6.3–9.5)	6.2 (5.7–7.2)	15.7 (13.3–20.4)	59.6^#^
			222	Placebo + chemotherapy^b^	45.4	4.3 (4.1–5.5)	5.4 (4.4–5.5)	11.1 (9.6–13.6)	56.4^#^
KEYVIBE-005 (E cohort)									
	All randomized patients	SCC 78% AC 22%	40	Vibostolimab/Pembrolizumab + chemotherapy^a^	52*	13.9 (7.9-NR)	10.4 (6.4–18.0)	18.0 (10.7-NR)	70.0^#^

AC, adenocarcinoma; SCC, squamous cell carcinoma; CI, confidence interval; HR, hazard ratio; No, number; OS, overall survival; PFS, progression free survival; DoR, duration of response; NR, not reached; AE, adverse events

^a^ Chemotherapy regimen in MORPHEUS-EC and KEYVIBE-005 (E cohort) were cisplatin (80 mg/m^2^, day 1) plus 5-fluorouracil (800 mg/m^2^, day 1 to 5) every 3 weeks; ^b^ SKYSCRAPER-08 employed paclitaxel (175 mg/m^2^, day 1) and cisplatin (60–85 mg/m^2^, day 1) every 3 weeks; * ESCC subgroup

^#^ Specified as treatment -related adverse events Note: In the KEYVIBE-005 (E cohort), one patient had a grade 5 TRAE of pneumonitis. In SKYSCRAPER-08, grade 5 TRAEs in the tiragolumab + atezolizumab + chemotherapy arm included immune-mediated lung disease, pneumonitis, cardiac arrest, gastrointestinal hemorrhage, hepatic failure, and bacterial pneumonia (n = 1 each), while in the placebo + chemotherapy arm, they included gastrointestinal infection and death (n = 1 each). Treatment-related adverse events were not specified in the MORPHEUS-EC presented poster

The KEYVIBE-005 study, an open-label phase 2 trial, investigated the effect of vibostolimab (an anti-TIGIT antibody) coformulated with pembrolizumab (an anti-PD-1 antibody) combined with cisplatin and 5-FU chemotherapy in patients with advanced EC who were candidates for first-line systemic therapy (cohort 5). Of the 40 enrolled patients, 31 (77.5%) had ESCC. The combination of vibostolimab and pembrolizumab with chemotherapy achieved an ORR of 53%, with a median duration of response of 13.9 months. The median PFS was 10.4 months, and the median OS was 18.0 months for the entire cohort of patients with EC (Table 3). The treatment regimen of vibostolimab and pembrolizumab plus chemotherapy was associated with a manageable safety profile. [33]

## Unanswered questions for the development of TIGIT-targeting therapy in ESCC

Clinical trial results for anti-TIGIT antibodies have shown that the combination of anti-TIGIT with anti-PD-1/PD-L1 was associated with clinical activity in previously treated patients with ESCC. Furthermore, the addition of this combination to platinum-based chemotherapy demonstrated promising antitumor efficacy as a first-line therapy in patients with advanced ESCC (Tables 2 and 3). However, the optimal role of anti-TIGIT therapy—whether as first-line or late-line treatment for ESCC—remains unclear. Well-designed phase III clinical trials incorporating the most current standard of care as a comparative arm are necessary to answer these questions.

In addition, three key areas of anti-TIGIT therapy require further investigation in ESCC. First, identifying clinically applicable biomarkers for anti-TIGIT ICI therapy is crucial. Second, the roles of anti-TIGIT ICI therapy in locoregional ESCC need to be explored. Third, gaining mechanistic insights and evaluating the clinical activities of various types of anti-TIGIT antibodies are essential.

### Predictive biomarkers for anti-TIGIT antibody and its combination

The biomarker studies for the anti-TIGIT program have primarily focused on PD-L1 expression levels. Although this approach is promising, it alone is insufficient. For instance, a comprehensive biomarker analysis from the phase 2 CITYSCAPE trial, which compared tiragolumab plus atezolizumab vs. atezolizumab alone as first-line treatment for non-small-cell lung cancer (NSCLC), found that intratumoral macrophages and serum macrophage activation were associated with clinical benefits in patients receiving the combination treatment [23]. High PD-L1 expression (i.e., tumor proportion score ≥ 50% on IHC staining) was correlated with a better ORR in the combination treatment arm compared with atezolizumab plus placebo. However, high levels of TIGIT or PVR, as determined through IHC staining, were not associated with improved clinical outcomes in patients receiving the combination treatment [34, 35]. An exploratory biomarker analysis from the SKYSCRAPER-04 study, which investigated the clinical activity of tiragolumab plus atezolizumab vs. atezolizumab alone in PD-L1 expressing cervical cancer, indicated that the low PD-L1 expression subgroup showed numerical improvements in PFS and OS in the combination treatment arm compared with the atezolizumab monotherapy arm. [32]

In ESCC trials, the combination of vibostolimab coformulated with pembrolizumab has demonstrated promising clinical activity in patients with ESCC without biomarker preselection. However, patients with high PD-L1 expression, defined by a combined positive score (CPS) of ≥ 10, had a higher ORR. [33]

Previous studies have demonstrated a coordinated expression of PD-L1 and TIGIT in tumor tissues. Thus, future studies should explore biomarkers that represent TIGIT/PVR signaling activity in tumor tissues along with PD-L1 expression levels. Moreover, biomarkers related to myeloid cells and Treg cells, which have been shown to mediate mechanisms contributing to the efficacy of anti-TIGIT therapy, also merit further research. [23]

### Clinical development of anti-TIGIT antibody in locoregional disease

The clinical development of anti-TIGIT therapy has predominantly focused on patients with advanced-stage solid cancers. However, CD155 (or PVR) and its related PVR/nectin family members, which are expressed on NK cells, play a vital role in the early stages of cancer elimination and in preventing metastasis [36]. Several ongoing studies are investigating the potential role of anti-TIGIT therapy in the earlier stages of various cancer types.

The development of anti-TIGIT ICI therapy for locoregional ESCC faces challenges due to the variety of established standard-of-care options. According to the ESMO Clinical Practice Guidelines for esophageal cancer, both definitive CRT and neoadjuvant CRT are recommended. [37] Recently, the JCOG1109 study, a phase III trial conducted in Japan, explored neoadjuvant chemotherapy or CRT for patients with locally advanced ESCC. The results indicated that neoadjuvant chemotherapy using a triplet regimen of docetaxel, cisplatin, and 5-FU was associated with significantly improved OS [38]. This finding suggests that neoadjuvant multi-drug combination chemotherapy may become another standard treatment option for patients with locoregional ESCC. The results of the MORPHEUS trial showed that adding tiragolomab to chemotherapy and atezolizumab improved response rates without increasing toxicity, suggesting that this combination may be promising as a neoadjuvant chemotherapy.

Multiple phase II and III studies have been initiated to explore the integration of anti-PD-1/PD-L1 ICIs in the treatment of locoregional ESCC. To date, the only clear benefit of adding anti-PD-1/PD-L1 ICIs to standard-of-care treatments has been observed with the use of adjuvant nivolumab in patients with locally advanced ESCC who have received neoadjuvant CRT [39]. Currently, the ongoing phase III SKYSCRAPER-07 study (NCT04543617) is investigating the role of anti-TIGIT ICI treatment in this patient group. This trial has enrolled patients who have undergone definitive CRT and randomized them to receive consolidation therapy with a dual ICI regimen of tiragolumab plus atezolizumab, atezolizumab alone, or placebos. The results are expected to provide valuable insights into the potential of anti-TIGIT therapy in the treatment of locally advanced ESCC [40]. Furthermore, a phase 1b/2 study in Taiwan (NCT05743504) is assessing the efficacy of neoadjuvant tiragolumab and atezolizumab combined with CRT followed by surgery in this patient population.

### Different anti-TIGIT antibodies

Multiple anti-TIGIT antibodies are currently undergoing clinical trials (Table 4). There is ongoing debate regarding the importance of Fc binding in the activity of these antibodies. Thus, clinical trials are exploring various designs of the Fc portion—Fc-competent, Fc-silent, and Fc-enhanced. A preclinical study demonstrated that combining Fc-enhanced or Fc-silent anti-TIGIT with anti-PD-1 in mice enhanced tumor control through distinct mechanisms: Fc-enhanced anti-TIGIT depleted intratumoral Treg, whereas Fc-silent anti-TIGIT enhances the activation of tumor-specific exhausted CD8 + T cells in a lymph node-dependent manner [41]. Fc-enhanced anti-TIGIT induced antibody-dependent cell-mediated cytotoxicity (ADCC) against human Tregs in vitro, and significant reductions in Tregs were observed in the peripheral blood of patients treated with AB308, an Fc-enhanced anti-TIGIT. On the other hand, Fc silent anti-TIGIT, Domvanalimab did not deplete human Treg in vitro and was associated with objective clinical responses while peripheral Treg frequencies remained stable on treatment [41]. Moreover, a preclinical study of tiragolumab, an Fc-competent anti-TIGIT antibody, demonstrated that it remodeled the tumor microenvironment by engaging IgG Fc receptors in an animal model, highlighting the potential clinical impact of Fc portion design on clinical outcomes [23]. In another preclinical study, ociperlimab, another Fc-competent anti-TIGIT antibody, enhanced T-cell functions, induced ADCC against Treg cells, activated NK cells and monocytes, and removed TIGIT from T-cell surfaces in an Fc-dependent manner [42]. This diversity in antibody engineering approaches highlights the critical question of optimal Fc portion design, which could significantly affect the efficacy and safety of anti-TIGIT therapies in treating ESCC.

**Table 4:** TIGIT-targeting antibodies under clinical development

	Anti-TIGIT antibodies	Antibody subtypes	Clinical development in EC
Fc-intact (Fc-competent)	Tiragolumab	IgG1 mAb	GO30103 (P1S, EC cohort included) MORPHEUS-EC (RP2S, ESCC and EAC) SKYSCRAPER-08 (P3S, ESCC) SKYSCRAPER-07 (P3S, ESCC)
	Vibostolimab	IgG1 mAb	KEYVIBE-005 cohort E (P2S, ESCC)
	Ociperlimab	IgG1 mAb	AdvanTIG-105 (P1S) AdvanTIG-203 (RP2S, ESCC)
Fc-silent	Domvanalimab	IgG1 mAb	STAR-221 (P3S, EAC) EDGE-Gastric (P2S, EAC)
	BMS-986207	IgG1 mAb targeting PVRIG and TIGIT	NCT04570839 (P1S, P2S; solid tumor) CA020-002 (P1S, P2S; solid tumor)
	Rilvegostomig (AZD2936)	IgG1 bispecific Ab targeting PD-1 and TIGIT	GEMINI-Gastric (P2S, EAC) DESTINY-Gastric03 (P2S, EAC)
Fc-enhanced	AB308	IgG1 mAb	NCT04772989 (P1S; solid tumor, EC dose expansion cohort included)
	AGEN1777 (BMS-986442)	Bispecific Ab targeting TIGIT and CD96	NCT05025085 (P1S; solid tumor)

*Fc portion* fragment crystallizable region, *mAb* monoclonal antibody, *ESCC* esophageal squamous cell carcinoma, *EAC* esophageal adenocarcinoma, *PD-1* programmed cell death protein-1, *PVRIG* poliovirus receptor-related immunoglobulin, *TIGIT* T-cell immunoreceptor with Ig and ITIM domains, *P1S* phase 1 study, *RP2S* randomized phase 2 study, *P3S* phase 3 study

## Conclusion

The exploration of anti-TIGIT agents in advanced ESCC has demonstrated promising potential, especially when combined with anti-PD-1/PD-L1 therapy, across various stages including late-line and first-line settings. However, to definitively establish the role of anti-TIGIT ICI treatments in ESCC, additional clinical trials that incorporate current standard-of-care treatments are necessary. Ongoing research into predictive biomarkers for anti-TIGIT ICI and the development of anti-TIGIT antibodies with optimal immunologic activity and therapeutic efficacy are critical for advancing TIGIT-targeting therapies in the treatment of ESCC and other cancers.

## Supplementary Information

Below is the link to the electronic supplementary material.

Supplementary file1 (DOCX 17 KB)
