# Supplementary material for: Exploring novel immunotherapy in advanced esophageal squamous cell carcinoma: Is targeting TIGIT an answer?
Source: Esophagus. 2025 Jan 23;22(2):139–47. doi: 10.1007/s10388-024-01105-4 (PMC11929690; doi:10.1007/s10388-024-01105-4)
Supplement: Supplementary file 1 — Supplementary file1 (DOCX 17 KB) [file 10388_2024_1105_MOESM1_ESM.docx]

| **Table S1 Key Results of Trials of Anti-TIGIT Monotherapy as Late-Line Systemic Therapy for Solid Tumors** | | | | | | | |
| --- | --- | --- | --- | --- | --- | --- | --- |
| **Trial** | **Population** | **P’t**  **No.** | **Treatment** | **ORR (%)** | **DCR (%)** | **Common TRAE (n, %)** | **Note** |
| **Anti-TIGIT monotherapy (dose escalation)** | | | | | | | |
| **GO30103** | | | | | | | |
|  | **Solid tumor patients** | 24 | Tiragolumab | 0 | 16.7 | fatigue (5, 21%), pruritus (3,13%), arthralgia (2, 8%): only grade 1 and 2 | 8% had received prior ICI 4% had grade 3 TRAE of increased creatinine |
| **MK-7684-001** | | | | | | | |
|  | **Solid tumor patients** | 34 | Vibostolimab | 0 | 32 | fatigue (5,15%), pruritus (5, 15%), nausea (3, 9%), and rash (3, 9%) | 9% had grade 3-4 TRAE |
|  | **Anti-PD-1/PD-L1 refractory NSCLC** | 34 | Vibostolimab | 3 | 3 | fatigue (7,21%), rash (7, 21%), arthralgia (4, 12%), and pruritus (3, 9%) | 15% had grade 3-4 TRAE |
| No grade 5 TRAEs were reported in either of the two trials mentioned above. Abbreviation: No, number; TIGIT, T cell immunoreceptor with Ig and ITIM domains; ORR, objective response rate; DCR, disease control rate; TRAE, treatment-related adverse events; ICI: immune checkpoint inhibitor; PD-1/PD-L1, programmed cell death protein 1//programmed cell death ligand 1 | | | | | | | |

Supplementary Table 1. Key Results of Trials of Anti-TIGIT Monotherapy as Late-Line Systemic Therapy for Solid Tumors
